# Non-Contact Respiratory Measurement Using a Depth Camera for Elderly People

**DOI:** 10.3390/s20236901

**Published:** 2020-12-03

**Authors:** Wakana Imano, Kenichi Kameyama, Malene Hollingdal, Jens Refsgaard, Knud Larsen, Cecilie Topp, Sissel Højsted Kronborg, Josefine Dam Gade, Birthe Dinesen

**Affiliations:** 1 Biomedical Engineering Laboratories, Teijin Pharma Ltd., Tokyo 191-8512, Japan; k.kameyama@teijin.co.jp; 2 Cardiology Ward, Regional Hospital Viborg, 8800 Sondersoparken, Denmark; malene.hollingdal@midt.rm.dk (M.H.); Jens.Refsgaard@viborg.rm.dk (J.R.); 3 Laboratory of Welfare Technology-Telehealth and Telerehabilitation, Department of Health Science and Technology, Aalborg University, 9220 Aalborg Ost, Denmark; knl@hst.aau.dk (K.L.); csrt@hst.aau.dk (C.T.); sisselhk@hst.aau.dk (S.H.K.); jdg@hst.aau.dk (J.D.G.); bid@hst.aau.dk (B.D.)

**Keywords:** tidal volume, respiratory rate, depth camera, respiratory motion

## Abstract

Measuring respiration at home for cardiac patients, a simple method that can detect the patient’s natural respiration, is needed. The purpose of this study was to develop an algorithm for estimating the tidal volume (TV) and respiratory rate (RR) from the depth value of the chest and/or abdomen, which were captured using a depth camera. The data of two different breathing patterns (normal and deep) were acquired from both the depth camera and the spirometer. The experiment was performed under two different clothing conditions (undressed and wearing a T-shirt). Thirty-nine elderly volunteers (male = 14) were enrolled in the experiment. The TV estimation algorithm for each condition was determined by regression analysis using the volume data from the spirometer as the objective variable and the depth motion data from the depth camera as the explanatory variable. The RR estimation was calculated from the peak interval. The mean absolute relative errors of the estimated TV for males were 14.0% under undressed conditions and 10.7% under T-shirt-wearing conditions; meanwhile, the relative errors for females were 14.7% and 15.5%, respectively. The estimation error for the RR was zero out of a total of 206 breaths under undressed conditions and two out of a total of 218 breaths under T-shirt-wearing conditions for males. Concerning females, the error was three out of a total of 329 breaths under undressed conditions and five out of a total of 344 breaths under T-shirt-wearing conditions. The developed algorithm for RR estimation was accurate enough, but the estimated occasionally TV had large errors, especially in deep breathing. The cause of such errors in TV estimation is presumed to be a result of the whole-body motion and inadequate setting of the measurement area.

## 1. Introduction

Cardiovascular disease (CVD) is the leading cause of death around the world, with approximately 17.9 million deaths each year according to the World Health Organization [1]. Heart failure (HF) is one of the outcomes of CVD, and it is estimated that there are approximately 26 million cases of HF worldwide [2]. HF is a clinical syndrome, which occurs when the heart is unable to provide enough blood flow to the body. HF is associated with repeated hospitalization caused by acute exacerbation, which strains the healthcare economy and decreases the patient’s quality of life (QoL).

One of the solutions to this problem is to use telemonitoring. According to a study by Bernocch et al. evidence from randomized trials confirms that telecommunication technologies have the best outcome in terms of prolonged survival and reduced hospital readmission rates for patients with HF [3]. Thus, self-monitoring and telecommunication could be beneficial approaches to detect the early warning signals of HF symptoms, although it can be difficult for elderly people to detect and recognize the symptoms by themselves [4]. Therefore, to make telemonitoring as efficient as possible, new measurement technologies should be available.

We focused on the assessment of dyspnea, as this is the most common symptom of HF and is, along with lung gas exchange and control of ventilation, now recognized as a meaningful indicator of disease severity and prognosis [5,6,7]. A study by Capucci et al. measured the breathing pattern of 528 HF patients and concluded that the respiratory rate and the rapid shallow breathing index changed significantly before an HF event. This suggests that these measures might be useful in the early identification of worsening HF status [8]. Thus, it could be beneficial for an HF patient to measure their tidal volume (TV) and respiratory rate (RR) daily to be able to detect the worsening of symptoms. However, it is not easy for a patient to use measuring devices for spirometry, impedance pneumography, or inductance plethysmography correctly, because they are primarily designed for clinical or research centers. Hence, they are not applicable for everyday use for home monitoring due to the complexity of the devices, their high cost, their need for skilled operators, and, in some cases, their limited portability [9,10]. Additionally, these devices require the user to be in direct contact with the equipment in an obtrusive manner, which interferes with natural respiration [10].

Therefore, to measure respiration at home for an HF patient, a simple method that can detect the patient’s natural respiration is urgently needed. Our research aimed to develop a system that can measure the TV and RR in the natural respiration of HF patients unobtrusively. We developed this system across multiple steps and started by testing the system on healthy elderly patients and then on cardiac patients.

Several sorts of contactless motion sensors can be used for the measurement of respiration. For example, a microwave Doppler sensor [11], a light coding 3D sensor [12], and a single camera [13] were used for sensing the TV and the RR in previous studies. Reyes et al. developed a TV measuring device by using a smartphone calibrated with an incentive spirometer (IS) that is commercially available. They tested this setup on 12 healthy subjects, who had to perform some different breathing patterns through the IS in front of a smartphone, which detected chest movements. The study concluded that the smartphone and IS slightly underestimated the respiration, but that they had a lot of advantages, such as being simple, fast, and affordable [9]. We used Microsoft Kinect for Xbox One (Microsoft Corp., WA, USA), a 3D Time-of-Flight (ToF) depth camera, because it is for home use and is a commercially available sensor that can precisely record the 3D motion of a non-rigid object such as a human body.

There are several studies related to our work. For example, the study by Aoki and Nakamura used a Kinect sensor to obtain the volume change due to respiration from six young male volunteers during an exercise stress test. The calculated respiration based on the sensor was compared to an expiratory gas analyzer, and the results suggested that by setting a region of interest (ROI) on the chest and abdomen, it was possible to calculate the respiration [14]. The study by Seppänen et al. aimed to estimate the respiratory airflow waveforms with a novel calibration method through a Kinect depth camera. Eight subjects were included in the trial, in which they performed different breathing styles while being measured using a depth camera and spirometer. The study concluded that it was possible to measure the TV and the RR very accurately [10].

However, most of the previous works did not study elderly subjects or HF patients. Moreover, subjects should be standing still or sitting with their back straight during measurements, but it is difficult for HF patients to maintain such a posture. Our system only requires that subjects sit on a chair naturally. The aim of this study was to develop an algorithm for the estimation of TV and RR using a 3D depth camera and to assess its accuracy for elderly subjects.

## 2. Methods

### 2.1. Ethical Aspects

This project was approved by the North Denmark Region Committee on Health Research Ethics (N-20190017) and the Danish Data Protection Agency, and was carried out following the Helsinki Declaration. All subjects signed an informed consent form before enrollment in the study.

### 2.2. Subjects

All of the subjects were 65 years of age or older, had no severe otolaryngitis, and had no history of heart failure or chronic obstructive pulmonary disease. All of the inclusion and exclusion criteria are shown in Table 1. All subjects were allowed to control the pace and depth of their breathing by themselves. Prior to the commencement of the experiment, the purpose of the study was explained verbally to the subject and written consent was obtained.

### 2.3. Experimental Setup and Measurement Procedure

**Figure 1:**
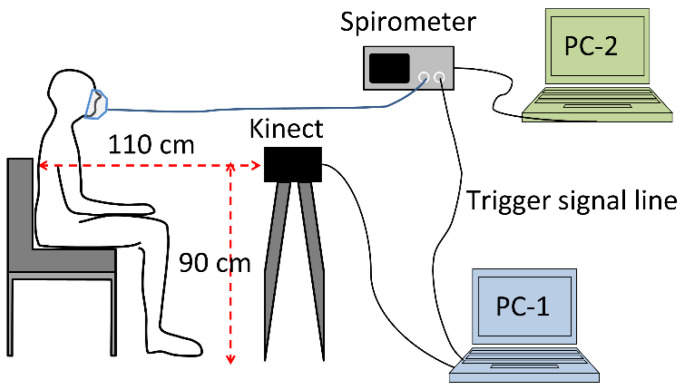
System configuration.

Figure 1 shows the experimental system configuration. A Microsoft Kinect for Xbox One (Kinect) was used as a 3D depth camera. The Kinect was connected to a Windows PC (PC-1: Dell Alienware 15-R2, Windows10 Home (64 bit)) via a Kinect Adapter for Windows. As a spirometer, a pneumotachograph SmartLab Data Acquisition System (Hans Rudolph, Inc., KS, USA) connected to a Windows PC (PC-2: Dell Latitude E5430) was used. To synchronize the measurement in both the Kinect and the spirometer, the software for Kinect on PC-1 sent a trigger signal of the Kinect measurement time to the spirometer via an analog line. Therefore, the Kinect measurement time was recorded in the spirometer data.

Before the measurements, the procedure was explained to the subject by a project nurse and the subject signed informed consent. His/her weight, height, chest circumference, and abdominal circumference were recorded. The subject was instructed to sit in front of the Kinect (Figure 1). The face mask for the spirometer was placed over the subject’s mouth. The TV was recorded by the spirometer. The depth images and the initial infrared (IR) image captured by the Kinect were recorded while the subject was breathing through the spirometer.

The subject was told to perform two different types of breathing patterns: (a) Normal breathing for 1 min and (b) five deep breaths at his/her own pace. The experiments were conducted under two different clothing conditions: undressed and in a T-shirt.

### 2.4. Estimation Method

**Figure 2:**
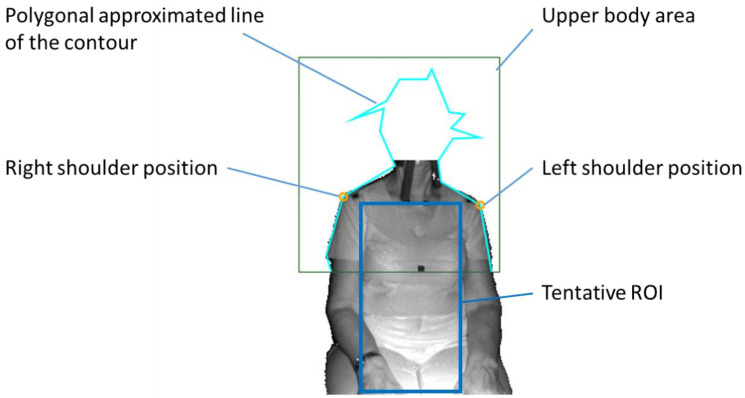
The tentative region of interest (ROI).

**Figure 3:**
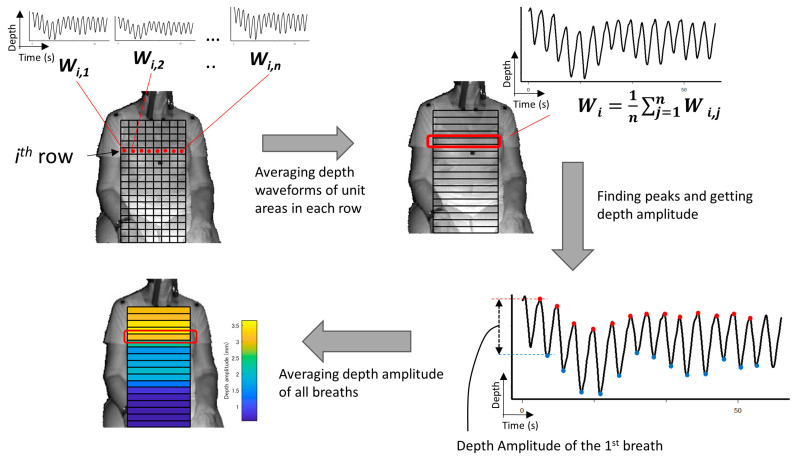
Acquiring the depth amplitude of each row.

**Figure 4:**
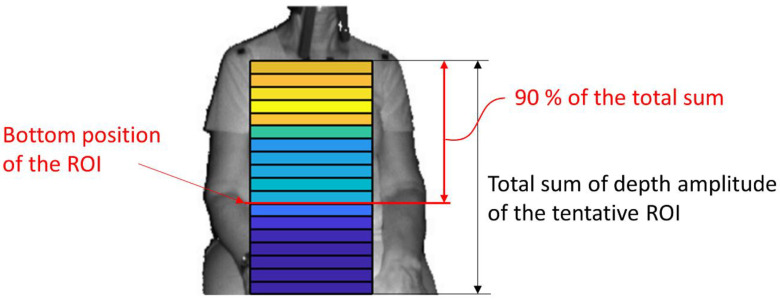
Determination of the proper bottom position of the ROI.

Since depth motion in breathing is found both in the chest and/or the abdomen, the ROI was set as a rectangle area passing through the subject’s shoulders and abdomen. The procedure for ROI determination consisted of the following steps (Figure 2, Figure 3 and Figure 4):

1. The detection of the upper body area: Haar cascade classifier in OpenCV [15] was used.
2. The detection of the position of both shoulders: Since the subject was sitting on a chair and had his/her arms lowered, the shoulder positions could easily be detected as the vertices in the polygonal approximated line of the contour of the upper body. We did not use the body tracking function in the Kinect for Windows SDK (Kinect SDK) [16] and also did not use a pose estimation library such as OpenPose [17]. This is because Kinect SDK is sometimes unstable when a subject is sitting, or because the pose estimation library requires a powerful computer with a graphics processing unit (GPU).
3. The determination of the tentative ROI: An example of a tentative ROI is shown as the blue rectangle area in Figure 2. The top of the ROI was set at the lowest position of the two shoulders. The left and right ends of the ROI were at the position corresponding to the inside the approximate arm’s width from both shoulders. The bottom position of the ROI was tentatively decided as the lower end of the image. Here, we employed a unit area of 19 × 19 pixels to reduce the random error of the depth measurements. Since there was a strong correlation among the depth values of close pixels [18], the size of the unit was decided by trial and error. The units that covered the tentative ROI area were slid every 10 pixels to acquire the respiratory signal. The obtained depth signals in each unit area were sampled at a rate of 30 Hz and were averaged in the area.
4. The determination of the final ROI: Since there is a difference in the contribution of the motion of the thoracic compartment or the abdomen to TV among sex and ages [19], we developed a unique algorithm to obtain the proper position of the bottom of the ROI. The algorithm was as follows:
  (A) The depth waveform of each row (*W_i_*) was calculated by averaging all of the depth waveforms (*W_i,*1*_*, *W_i,*2*_*, …, *W_i,n_*) of the unit areas in each row. The depth amplitude of each row was calculated by averaging the depth amplitude of each breath in waveform *W_i_* (Figure 3).
  (B) The amplitude ratio of each row was calculated from the sum of the depth amplitude of all rows.
  (C) The amplitude ratio was integrated from the top of the ROI, and the position where it reached 90% was defined as the bottom of the ROI (Figure 4). A value of 90% was decided by trial and error.

**Figure 5:**
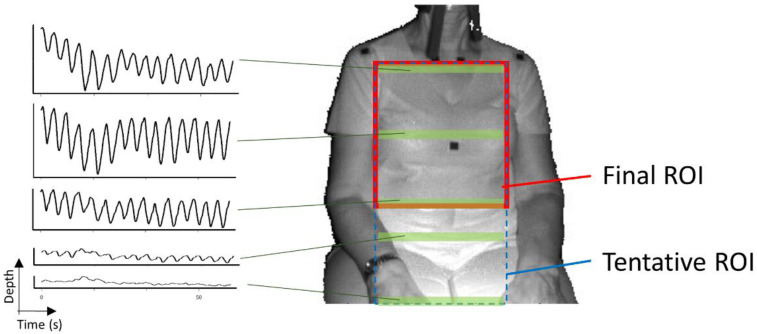
Final ROI, tentative ROI, and some of the depth waveforms.

Figure 5 shows the final ROI (red solid box), the tentative ROI (blue dashed line box), and the depth waveforms in some rows (green area). The depth waveforms in the final ROI are much clearer than that outside of the final ROI. This means that the algorithm can properly omit the inadequate area in the tentative ROI.

**Figure 6:**
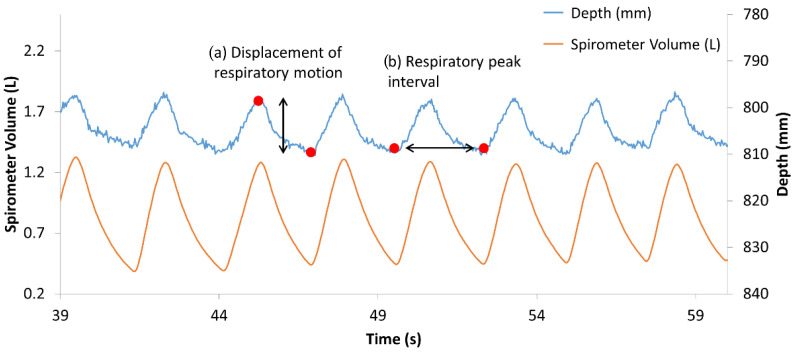
Data captured from the spirometer volume and depth camera.

Once the ROI was set, the depth signal of the ROI was averaged, resampled with a rate of 30 Hz, and smoothed. The depth amplitude, namely, the displacements of respiratory motion, were obtained by finding the peaks and the valleys from the processed depth signal (Figure 6a). The volume data from the spirometer were also processed in the same way to calculate the TV. The TV estimation equation was obtained by single linear regression analysis using the TV from the spirometer as the objective variable and the amplitude from the depth camera as the explanatory variable. Concerning the RR estimation, the time for each respiration was calculated from the peak interval (Figure 6b). The estimated RR for each breath (breaths per minute (BPM)) was its reciprocal value and compared to the value from the spirometer.

### 2.5. Statistical Analysis

Bland–Altman analysis [20] was performed on the spirometer volume and the estimated TV to calculate the 95% confidence intervals (95% CIs) and the Pearson correlation, which were used to evaluate the presence of constant bias and proportional bias. The significance level was 5%. The mean absolute relative error against the spirometer volume was calculated to evaluate the estimation error. Concerning the estimation error in RR, the error was counted if the difference between the corresponding RRs exceeded 1 bpm. Then, the frequency at which the error appeared was used as the estimation error. Microsoft Excel (2016) (Microsoft Corp., WA, USA) was used for statistical analysis.

## 3. Results

Table 2 shows the baseline characteristics of the subjects.

**Figure 7:**
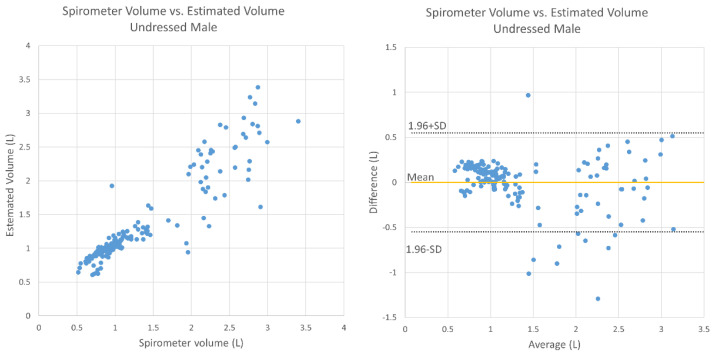
Bland–Altman plot for TV obtained using the spirometer and the estimated TV under undressed conditions (male subjects).

**Figure 8:**
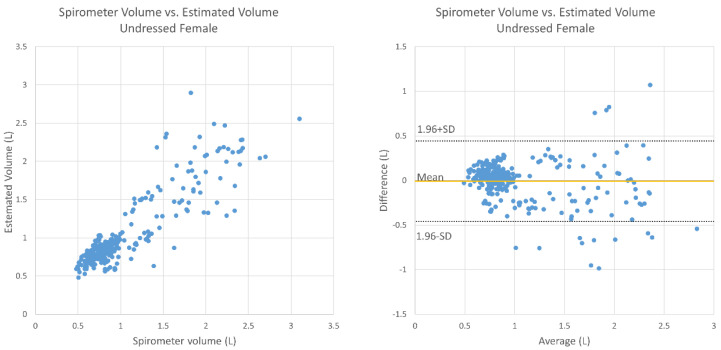
Bland–Altman plot for TV obtained using the spirometer and the estimated TV under undressed conditions (female subjects).

Figure 7 shows the relationship between the estimated TV and the TV obtained by the spirometer under undressed conditions for the male subjects. The mean absolute relative error was 14.0%, there was no fixed bias (95% CI, −0.044 to 0.084), and there was proportional bias (correlation, −0.209; *p* < 0.05). Figure 8 shows a graph for the female subjects under undressed conditions; the average relative error was 14.7%, there was no fixed bias (95% CI, −0.035 to 0.045), and there was proportional bias (correlation, −0.244, *p* < 0.05).

**Figure 9:**
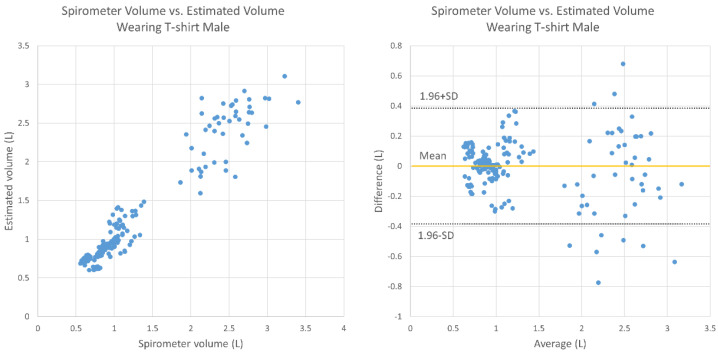
Bland–Altman plot for TV obtained using the spirometer and the estimated TV under T-shirt-wearing conditions (male subjects).

**Figure 10:**
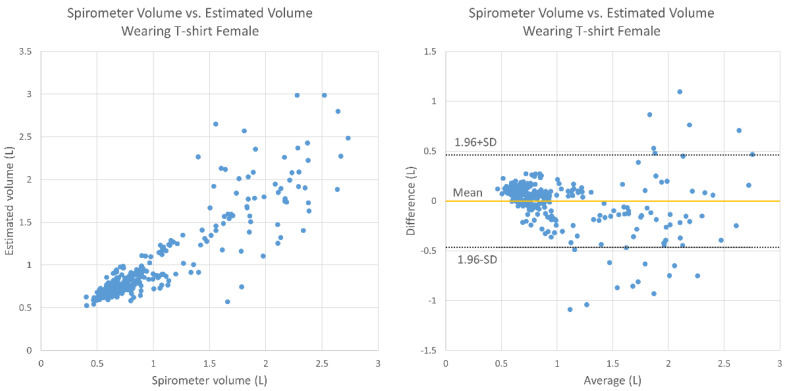
Bland–Altman plot for TV obtained using the spirometer and the estimated TV under T-shirt-wearing conditions (female subjects).

Figure 9 and Figure 10 display the results under T-shirt-wearing conditions for the male and female subjects, respectively. For the male subjects, the average relative error was 10.7%, there was no fixed bias (95% CI, −0.030 to 0.067), and there was no proportional bias (correlation, −0.136; *p* > 0.05). For the female subjects, the average relative error was 15.5%, there was no fixed bias (95% CI, −0.028 to 0.053), and there was proportional bias (correlation, −0.239; *p* < 0.05).

The estimation error for the RR was zero out of a total of 206 breaths under undressed conditions and two out of a total of 218 breaths under T-shirt-wearing conditions for the males. Concerning the females, the error was three out of a total of 329 breaths under undressed conditions and five out of a total of 344 breaths under T-shirt-wearing conditions.

## 4. Discussion

This study mainly aimed to test and evaluate the functions of TV and RR estimation using the Kinect camera on healthy elderly subjects. There were weak negative correlations in breathing conditions except for the case of males wearing a T-shirt. In the Bland–Altman plots (Figure 7, Figure 8, Figure 9 and Figure 10), the vertical distribution spread toward the right, which means that the TV estimation of deep breathing included more errors than normal breathing. Furthermore, in most of the cases, the errors were caused by the underestimation of TV. There are two possible reasons for this: (1) the motion of bending backward on exhalation timing in deep breathing, which leads to a smaller depth value because the backward motion of the whole body cancels the chest motion of exhalation; (2) the motion asynchronization between the chest and the abdomen in some subjects. In such cases, the developed algorithm estimated a smaller TV than when the chest and the abdomen were synchronously moved.

In the software used in the experiment, the ROI for obtaining the depth signal was set only at the beginning of the measurement. However, the fixed ROI setting was not suitable when the subject breaths deeply. This is because the chest and the abdomen might have been moving not only in the posterior or anterior direction but also in the other directions in such cases. At the time of exhalation during deep breathing, there were some cases where the upper part of the body was omitted. Aoki et al. proposed a method for extracting the motion of the parts of the body (chest and abdomen) likely to exhibit respiratory movements using the respiratory cycle. The results showed that by using the thoraco-abdominal region, it is possible to improve the accuracy of quasi-tidal volume variation rather than using the entire upper body [21]. Their other study reported that by updating the ROI frame by frame, breathing can be measured in a non-contact manner even with large body movements during pedaling [14]. In our algorithm, the determination of ROI is considered to contribute greatly to the estimation accuracy. In this experiment, there was not body movement as much as pedaling motion, but it was necessary to update the ROI in every frame to take into account the body movement during deep breathing. Moreover, in our current algorithm, is assumed that there is not much difference in the motion and shape between the left and the right side of the thorax and abdomen. Therefore, our method cannot be applied to subjects with imbalanced thoracic motion such as pectus excavatum. For such subjects, different methods will be necessary [22].

In this study, the error under undressed conditions was larger than the error under clothed conditions in the case of male. This might have been caused by vertical body movement. Since there is large unevenness in the chest and abdomen under undressed conditions compared to that under clothed conditions, vertical body motion might cause noise in the measurement. This might have also caused the RR errors. If the uneven part of the body moves not only in a depth direction but also in a vertical direction in each breath, the phase of the breathing waveform may not be stable. Therefore, it is necessary to correct the respiratory motion by measuring the horizontal and vertical movements. This research, however, is subject to a couple of limitations:

- The deep breathing outlier data could not be omitted since we set the minimum number of deep breaths as five in the protocol. This might have affected the accuracy of the TV estimation since there was relatively large motion during the deep breathing.
- There was only one set of trials for each subject, meaning that we could not confirm the reproducibility of the measurements for one person, which is important for tracking the change of HF pathology. Thus, future research is needed.

## 5. Conclusions

The algorithm for estimating the TV and RR from the depth motion of the chest and/or the abdomen captured by a depth camera was developed for both undressed and T-shirt-wearing conditions. The accuracy of the estimated TV and RR was evaluated by comparison with those acquired using a spirometer. The experimental result showed that the RR estimation of our algorithm had a maximum error rate of 1.5% (five errors out of a total of 344 breaths), but the estimated TV occasionally had large errors, especially during deep breathing. Furthermore, we evaluated how much the presence or absence of clothing affected the estimation accuracy, and found that the error tended to be larger under undressed conditions than under T-shirt-wearing conditions. The cause of such errors is considered to be based on the ROI settings and the vertical motion in the chest and abdomen.

## Figures and Tables

**Table 1:** Inclusion and exclusion criteria.

Healthy Elderly People	
➢ Inclusion Criteria:	
	Age of 65–75 years
	No chronic cough
	No chronic sputum
	No exertional dyspnea
➢ Exclusion Criteria:	
	Previous neurologic, musculoskeletal, or mental illnesses
	Lack of ability to cooperate
	Silicon allergy
	Surgical history or currently under treatment for cardiopulmonary diseases
	Aurinasal disease
	Symptoms of nasal congestion (cannot breathe in the nose)
	Suspected digestive organ diseases, liver diseases, renal diseases, cardiovascular diseases, blood diseases, endocrine diseases, or malignant neoplasms, or a history of any such conditions
	Depressive symptoms or a depression diagnosis
	Bronchial asthma
	Diffuse panbronchiolitis
	Congenital sinus bronchial syndrome
	Bronchiolitis obliterans
	Bronchial ectasia
	Lung tuberculosis
	Pneumoconiosis
	Pulmonary lymphangioleiomyomatosis
	Cold-like symptoms on the day of the examination

**Table 2:** Baseline characteristics of the subjects.

	Male (*n* = 14)		Female (*n* = 25)	
	Avg.	SD	Avg.	SD
Age (years)	69.9	2.85	68.6	8.93
Height (cm)	179.3	6.01	166.7	4.06
Weight (kg)	87.2	12.10	71.1	15.34
Chest (cm)	103.5	10.20	87.4	13.37
Abdomen (cm)	105.6	11.83	93.4	16.19
